# The Real-Valued Sparse Direction of Arrival (DOA) Estimation Based on the Khatri-Rao Product

**DOI:** 10.3390/s16050693

**Published:** 2016-05-13

**Authors:** Tao Chen, Huanxin Wu, Zhongkai Zhao

**Affiliations:** College of Information and Communication Engineering, Harbin Engineering University, Harbin 150001, China; chentao@hrbeu.edu.cn (T.C.); wuhuanxin1990@163.com (H.W.)

**Keywords:** sparse direction of arrival (DOA) estimation, multiple measurement vectors (MMV), Khatri-Rao (KR) product, unitary transformation, array covariance vectors

## Abstract

There is a problem that complex operation which leads to a heavy calculation burden is required when the direction of arrival (DOA) of a sparse signal is estimated by using the array covariance matrix. The solution of the multiple measurement vectors (MMV) model is difficult. In this paper, a real-valued sparse DOA estimation algorithm based on the Khatri-Rao (KR) product called the L_1_-RVSKR is proposed. The proposed algorithm is based on the sparse representation of the array covariance matrix. The array covariance matrix is transformed to a real-valued matrix via a unitary transformation so that a real-valued sparse model is achieved. The real-valued sparse model is vectorized for transforming to a single measurement vector (SMV) model, and a new virtual overcomplete dictionary is constructed according to the KR product’s property. Finally, the sparse DOA estimation is solved by utilizing the idea of a sparse representation of array covariance vectors (SRACV). The simulation results demonstrate the superior performance and the low computational complexity of the proposed algorithm.

## 1. Introduction

The direction of arrival (DOA) estimation is an important area of research in array signal processing. Currently, there are many DOA estimation algorithms with good performance, such as the multiple signal classification (MUSIC) algorithm, the estimation of signal parameters via rotational invariance techniques (ESPRIT) algorithm, *etc.* Most of these traditional DOA estimation algorithms require the source number as *a priori* information and a large number of snapshots to guarantee the estimation precision. In recent years, the DOA estimation that utilizes the idea of sparse representation has become many scholars’ research hotspot [1,2]. The target signals can be regarded to be sparse in a spatial domain, and their DOAs can be estimated according to the array received data and a redundant dictionary. The DOA estimation algorithm by utilizing the idea of sparse representation mainly divides into two kinds [3,4]. One is the sparse model based on the array received data, and the other one is the sparse model based on the array covariance matrix. The singular value decomposition (SVD) of the array received data is used to propose a L_1_-SVD algorithm for DOA estimation [5]. Literature [6] introduces the idea of a sparse representation of array covariance vectors (SRACV) to propose a method called the L_1_-SRACV algorithm for estimating sparse signals’ DOAs. Compared to L_1_-SVD, the L_1_-SRACV algorithm does not need to determine the regularization parameter and has a higher stability. However, the L_1_-SRACV algorithm needs to estimate the noise power. In [7,8], L_1_-SRACV is improved such that the noise power is unnecessary and the algorithm’s robustness is enhanced. However, the above algorithms are all the resolution problems of the multiple measurement vectors (MMV) model and refer to complex operations and thus a heavy calculation burden. In this paper, the sparse model based on the array covariance matrix is transformed to a real-valued sparse model via a unitary transformation so that the amount of calculation is reduced by at least four times. Then, the real-valued sparse model is vectorized, and the MMV model is transformed to a single measurement vector (SMV) one whose calculation complexity is lower. Recently, the Khatri-Rao (KR) approach for DOA estimation increases rapidly. It can extend the array aperture effectively, increase the degree of freedom, and deal with the underdetermined DOA estimation in cases where the number of array elements is less than the source number in some conditions [9,10,11]. According to the property of the KR product and the real-valued overcomplete dictionary achieved by a unitary transformation, this paper constructs a new real-valued dictionary and uses the idea of a SRACV to estimate the target signals’ DOAs. The simulations demonstrate that the proposed algorithm has a good estimation performance and a low amount of calculation.

In this paper, the italic letters, the bold italic capital letters, and the bold italic lower-case letters denote variables, matrices, and vectors, respectively. The remainder of this paper is organized as follows. The sparse DOA estimation model based on the array covariance matrix is introduced in Section 2. The proposed algorithm and the detailed formula derivation of the proposed algorithm is given in Section 3. The simulation results and conclusions are given in Section 4 and Section 5, respectively.

## 2. Signal Model for DOA Estimation

### 2.1. Input Signal Model

Suppose a uniform linear array (ULA) which has M elements, and the element spacing is d. There are K uncorrelated far-field narrowband signals impinging on the array from directions $\theta =[\theta_{1}\;\theta_{2}\;\cdots \;\theta_{K}]$. The array received signal is given by

(1) y(t)=As(t)+n(t), t=1,2,⋯,L

where $A=[a(\theta_{1})\;a(\theta_{2})\;\cdots \;a(\theta_{K})]$ is the M×K array manifold matrix whose steering vector is $a(\theta_{k})={\left[1,\;e^{j\phi (\theta_{k})}\;,\cdots \;,e^{j(M-1)\phi (\theta_{k})}\right]}^{T}$, $\phi (\theta_{k})=-2\pi \frac{d}{\lambda }\sin(\theta_{k})$, λ denotes the wavelength of the incident signals, ${\left(\cdot \right)}^{T}$ denotes the transpose, s(t) is the Gaussian signal with mean zero, n(t) is additive Gaussian white noise, and L is the number of snapshots.

### 2.2. Signal Model Based on Sparse Representation

Suppose that the grid $\alpha ={\left[\alpha_{1},\alpha_{2},\cdots ,\alpha_{P}\right]}^{T}$ (P≫K) contains all the potential directions in the spatial domain. The sparse representation of the signal can be expressed as follows:

(2) $$Y=\bar{A}X+N$$

where $\bar{A}=[a(\alpha_{1})\;a(\alpha_{2})\;\cdots \;a(\alpha_{P})]$ is an overcomplete dictionary of size M×P, $X={\left[x_{1},x_{2},\cdots ,x_{P}\right]}^{T}$ is a sparse signal of size P×L in the spatial domain, and $x_{p}(t)$ is equal to $s_{k}\;(t)$ when the kth target locates at $\alpha_{p}$ and is zero otherwise.

The array covariance matrix is

(3) $$R_{Y}=E\{YY^{H}\}=\bar{A}R_{X}{\bar{A}}^{H}+\sigma^{2}I_{M}$$

where ${\left(\cdot \right)}^{H}$ denotes the conjugate transpose, $R_{X}=E\{XX^{H}\}$ is the covariance matrix of the sparse signal X where E{·} denotes the expectation, and $R_{X}=\mathrm{diag}\{\sigma_{1}^{2},\sigma_{2}^{2},\cdots ,\sigma_{P}^{2}\}$, $\sigma_{p}^{2}$ is the pth spatial signal’s power, $\sigma^{2}$ denotes the noise power, and $I_{M}$ denotes an identity matrix of size M×M.

## 3. The Real-Valued Sparse DOA Estimation Based on the KR Product

### 3.1. The Real-Valued Sparse Model for DOA Estimation

In this section, a real-valued sparse DOA estimation algorithm based on the KR product called the L_1_-RVSKR is proposed. As multiplication operation occupies the most proportion in the calculation and a complex multiplication requires four real number multiplications, the complex data of the array can be transformed to real data in order to reduce the computational complexity [12,13,14,15]. Define a unitary matrix as follows:

(4) $$Q=\left\{ \begin{array}{ll} \frac{1}{\sqrt{2}}\left[\begin{array}{lll} \;I{\;}_{\frac{M}{2}} & & J_{\frac{M}{2}}\\ jJ{\;}_{\frac{M}{2}} & & -jI_{\frac{M}{2}} \end{array}\right] & \mathrm{if}\;M\;\mathrm{is}\;\mathrm{even}\\ \frac{1}{\sqrt{2}}\left[\begin{array}{lllll} \;I{\;}_{\frac{M-1}{2}} & & 0_{\frac{M-1}{2}\times 1} & & J_{\frac{M-1}{2}}\\ \;0_{\frac{M-1}{2}\times 1}^{T} & & \sqrt{2} & & 0_{\frac{M-1}{2}\times 1}^{T}\\ jJ{\;}_{\frac{M-1}{2}} & & 0_{\frac{M-1}{2}\times 1} & & -jI_{\frac{M-1}{2}} \end{array}\right] & \mathrm{if}\;M\;\mathrm{is}\;\mathrm{odd} \end{array}\right.$$

where $I_{\frac{M}{2}}$, $J_{\frac{M}{2}}$, and $0_{\frac{M-1}{2}\times 1}$ denote the $\frac{M}{2}\times \frac{M}{2}$ identity matrix, the $\frac{M}{2}\times \frac{M}{2}$ permutation matrix, and the $\frac{M-1}{2}\times 1$ null matrix, respectively.

**Theorem 1.**  For any M×M Hermitian persymmetric matrix D, $QDQ^{H}$ is real and symmetric.

However, as the number of snapshots in practice is finite, the practical sampling covariance matrix is Hermitian but generally not persymmetric. Therefore, the persymmetrized estimator of the practical sampling covariance matrix is

(5) $$\begin{array}{ll} R & =\frac{1}{2}(R_{Y}+J_{M}R_{Y}^{*}J_{M})\\ & =\frac{1}{2}(\bar{A}R_{X}{\bar{A}}^{H}+J_{M}{\left(\bar{A}R_{X}{\bar{A}}^{H}\right)}^{*}J_{M})+\sigma^{2}I_{M}\\ & =\frac{1}{2}(\bar{A}R_{X}{\bar{A}}^{H}+(J_{M}{\bar{A}}^{*})R_{X}^{*}{\left(J_{M}{\bar{A}}^{*}\right)}^{H})+\sigma^{2}I_{M} \end{array}$$

where ${\left(\cdot \right)}^{*}$ denotes the complex conjugate.

The array manifold matrix of the isometric uniform linear array satisfies that $J_{M}{\bar{A}}^{*}=\bar{A}B$ and we have

(6) $$\begin{array}{cc} R & =\frac{1}{2}(\bar{A}R_{X}{\bar{A}}^{H}+\bar{A}BR_{X}^{*}B^{H}{\bar{A}}^{H})+\sigma^{2}I_{M}\\ & =\frac{1}{2}\bar{A}(R_{X}+BR_{X}^{*}B^{H}){\bar{A}}^{H}+\sigma^{2}I_{M} \end{array}$$

where the diagonal matrix $B=\Phi^{1-M}$, and the rotation matrix $\Phi =\mathrm{diag}\{e^{j\phi (\alpha_{1})}\;\cdots \;e^{j\phi (\alpha_{P})}\}$.

The real-valued symmetrical matrix can be achieved via a unitary transformation

(7) $$\begin{array}{cc} R_{r} & =QRQ^{H}\\ & =\frac{1}{2}Q\bar{A}(R_{X}+BR_{X}^{*}B^{H}){\bar{A}}^{H}Q^{H}+\sigma^{2}I_{M} \end{array}$$

The equation $B=\Phi^{1-M}$ is substituted into Equation (7) and we have

(8) Rr=12QA¯(RX+Φ1−MRX*ΦM−1)A¯HQH+σ2IM=12QA¯Φ1−M2ΦM−12(RX+Φ1−MRX*ΦM−1)Φ1−M2ΦM−12A¯HQH+σ2IM=12QA_ΦM−12(RX+Φ1−MRX*ΦM−1)Φ1−M2A_HQH+σ2IM=12QA_(ΦM−12RXΦ1−M2+(ΦM−12RXΦ1−M2)*)A_HQH+σ2IM=12Ψ(ΦM−12RXΦ1−M2+(ΦM−12RXΦ1−M2)*)ΨH+σ2IM=ΨRe(ΦM−12RXΦ1−M2)ΨT+σ2IM

where Re(·) denotes the real part and

(9) $$\underline{A}=\bar{A}\Phi^{\frac{1-M}{2}}=[\underline{a}(\alpha_{1})\;\underline{a}(\alpha_{2})\;\cdots \;\underline{a}(\alpha_{P})]$$

(10) $$\underline{a}(\alpha_{p})=\left\{ \begin{array}{ll} {\left[e^{-j\frac{M-1}{2}\phi (\alpha_{p})}\;,\cdots \;,e^{-j\frac{1}{2}\phi (\alpha_{p})},e^{j\frac{1}{2}\phi (\alpha_{p})}\;,\cdots ,e^{j\frac{M-1}{2}\phi (\alpha_{p})}\right]}^{T} & \mathrm{if}\;M\;\mathrm{is}\text{ even}\\ {\left[e^{-j\frac{M-1}{2}\phi (\alpha_{p})}\;,\cdots \;,1\;,\cdots ,e^{j\frac{M-1}{2}\phi (\alpha_{p})}\right]}^{T} & \mathrm{if}\;M\;\mathrm{is}\;\mathrm{odd} \end{array}\right.$$

and the real-valued overcomplete dictionary is

(11) $$\Psi =Q\underline{A}=[\psi (\alpha_{1})\;\psi (\alpha_{2})\;\cdots \;\psi (\alpha_{P})]$$

If M is even, the real-valued steering vector is

(12) $$\psi (\alpha_{p})=\sqrt{2}{\left[\cos(\frac{M-1}{2}\phi (\alpha_{p}))\;,\cdots \;,\cos(\frac{1}{2}\phi (\alpha_{p}))\;,\sin(\frac{1}{2}\phi (\alpha_{p}))\;,\cdots ,\sin(\frac{M-1}{2}\phi (\alpha_{p}))\;\right]}^{T}$$

and, if M is odd, the real-valued steering vector is

(13) $$\psi (\alpha_{p})=\sqrt{2}{\left[\cos(\frac{M-1}{2}\phi (\alpha_{p}))\;,\cdots ,\cos(\phi (\alpha_{p}))\;,1,\sin(\phi (\alpha_{p}))\;,\cdots ,\sin(\frac{M-1}{2}\phi (\alpha_{p}))\;\right]}^{T}$$

Then, an important property of the KR product is used with Equation (8). The property of the KR product can be expressed as follows [9]:

(14) $$\mathrm{vec}(ϒΖ\Xi^{H})=(\Xi^{*}⊙ϒ)\zeta$$

where vec(·) is the vectorization operation that stacks each column of a matrix one by one, $ϒ=[\upsilon_{1},\upsilon_{2},\cdots ,\upsilon_{k}]\in {\mathbb{C}}^{m\times k}$, $\Xi =[\xi_{1},\xi_{2},\cdots ,\xi_{k}]\in {\mathbb{C}}^{n\times k}$, ⊙ denotes the KR product, $\Xi^{*}⊙ϒ=[\xi_{1}^{*}⊗\upsilon_{1},\xi_{2}^{*}⊗\upsilon_{2},\cdots ,\xi_{k}^{*}⊗\upsilon_{k}]$, ⊗ denotes Kronecker product, $\zeta ={\left[\zeta_{1},\zeta_{2},\cdots ,\zeta_{k}\right]}^{T}$, and Ζ=diag(ζ) is a diagonal matrix.

According to the above property, Equation (14) is applied to Equation (8), and Equation (8) can be vectorized as

(15) rr=vec(Rr)=(Ψ⊙Ψ)·diag{Re(ΦM−12RXΦ1−M2)}+σ2vec(IM)=(Ψ⊙Ψ)·u+σ2vec(IM)

where $r_{r}=\mathrm{vec}(R_{r})$ is the vector form of $R_{r}$, and u=diag{Re(ΦM−12RXΦ1−M2)} is a vector which is consist of the diagonal elements of the matrix Re(ΦM−12RXΦ1−M2). Equation (15) can be considered a new observation model, in which the observation vector $r_{r}$, the dictionary (Ψ⊙Ψ), and the sparse vector u are all real-valued. Furthermore, the MMV model is transformed to a SMV model by using the KR product’s property. Therefore, the computational complexity can be effectively decreased. Moreover, the dictionary is constructed via the KR product so that a new virtual array is produced [16]. The dimension of the produced virtual array (Ψ⊙Ψ) is $M^{2}$, and the number of the distinct rows in (Ψ⊙Ψ) is 2M−1, which is greater than the matrix Ψ’s dimension M. Therefore, the array aperture is extended, and the resolving power is effectively improved.

### 3.2. DOA Estimation Based on a SRACV

According to Equations (5) and (7), $R_{r}$ can also be formulated as

(16) $$\begin{array}{ll} R_{r} & =\frac{1}{2}Q(R_{Y}+J_{M}R_{Y}^{*}J_{M})Q^{H}\\ & =\frac{1}{2}QR_{Y}Q^{H}+\frac{1}{2}QJ_{M}R_{Y}^{*}J_{M}Q^{H}\\ & =R_{r1}+R_{r2} \end{array}$$

where $R_{r1}=\frac{1}{2}QR_{Y}Q^{H}$ and $R_{r2}=\frac{1}{2}QJ_{M}R_{Y}^{*}J_{M}Q^{H}$.

The vector form of Equation (16) can be expressed as

(17) $$r_{r}=\mathrm{vec}(R_{r})=\mathrm{vec}(R_{r1})+\mathrm{vec}(R_{r2})$$

where

(18) $$\mathrm{vec}(R_{r1})=\frac{1}{2}\mathrm{vec}(QR_{Y}Q^{H})=\frac{1}{2}(Q^{*}⊗Q)\mathrm{vec}(R_{Y})=G_{1}\mathrm{vec}(R_{Y})$$

(19) $$G_{1}=\frac{1}{2}(Q^{*}⊗Q)$$

(20) $$\begin{array}{cc} \mathrm{vec}(R_{r2}) & =\frac{1}{2}\mathrm{vec}(QJ_{M}R_{Y}^{*}J_{M}Q^{H})\\ & =\frac{1}{2}(Q^{*}⊗Q)\mathrm{vec}(J_{M}R_{Y}^{*}J_{M})\\ & =\frac{1}{2}(Q^{*}⊗Q)(J_{M}⊗J_{M})\mathrm{vec}(R_{Y}^{*})\\ & =G_{2}\mathrm{vec}(R_{Y}^{*}) \end{array}$$

and

(21) $$G_{2}=\frac{1}{2}(Q^{*}⊗Q)(J_{M}⊗J_{M})$$

As the number of snapshots is finite in practice, the practical sampling covariance matrix is given by

(22) $${\hat{R}}_{Y}=\frac{1}{L}\sum_{t=1}^{L}YY^{H}=R_{Y}+\Delta R$$

where $\Delta R={\hat{R}}_{Y}-R_{Y}$ is the estimate error. The vector $\Delta r=\text{vec}(\Delta R)=\text{vec}({\hat{R}}_{Y}-R_{Y})$ satisfies an asymptotic normal distribution [17,18]

(23) $$\Delta r\sim \mathrm{AsN}(0_{M^{2}\times 1},\frac{1}{L}R_{Y}^{T}⊗R_{Y})$$

where $\text{AsN}(\mu ,\sigma^{2})$ denotes an asymptotic normal distribution whose expectation is μ and whose variance is $\sigma^{2}$. According to the literature [17,18,19], it can be known that

(24) $$\mathrm{vec}(\Delta R_{r1})\sim \text{AsN}(0_{M^{2}\times 1},G_{1}(\frac{1}{L}R_{Y}^{T}⊗R_{Y})G_{1}^{H})$$

and

(25) $$\mathrm{vec}(\Delta R_{r2})\sim \text{AsN}(0_{M^{2}\times 1},G_{2}(\frac{1}{L}R_{Y}⊗R_{Y}^{*})G_{2}^{H})$$

where

(26) $$\mathrm{vec}(\Delta R_{r1})=\mathrm{vec}(\frac{1}{2}Q{\hat{R}}_{Y}Q^{H}-\frac{1}{2}QR_{Y}Q^{H})=G_{1}\Delta r$$

and

(27) $$\mathrm{vec}(\Delta R_{r2})=\mathrm{vec}(\frac{1}{2}QJ_{M}{\hat{R}}_{Y}^{*}J_{M}Q^{H}-\frac{1}{2}QJ_{M}R_{Y}^{*}J_{M}Q^{H})=G_{2}\Delta r^{*}$$

In order to obtain the covariance of Δr and $\Delta r^{*}$, firstly we have

(28) $$\begin{array}{l} E\{{\hat{r}}_{i}{\left({\hat{r}}_{j}^{*}\right)}^{H}\}=\frac{1}{L^{2}}\sum_{t_{1}=1}^{L}\sum_{t_{2}=1}^{L}E\{y(t_{1})y_{i}^{*}(t_{1}){\left(y(t_{2})y_{j}^{*}(t_{2})\right)}^{T}\}\\ =\frac{1}{L^{2}}\sum_{t_{1}=1}^{L}\sum_{t_{2}=1,t_{2}\neq t_{1}}^{L}E\{y(t_{1})y_{i}^{*}(t_{1})\}E\{{\left(y(t_{2})y_{j}^{*}(t_{2})\right)}^{T}\}+\frac{1}{L^{2}}\sum_{t_{1}=1}^{L}E\{y(t_{1})y_{i}^{*}(t_{1})y_{j}^{*}(t_{1})y^{T}(t_{1})\}\\ =\frac{L^{2}-L}{L^{2}}r_{i}r_{j}^{T}+\frac{1}{L}(r_{i}r_{j}^{T}+r_{j}r_{i}^{T})\\ =(1-\frac{1}{L})r_{i}r_{j}^{T}+\frac{1}{L}(r_{i}r_{j}^{T}+r_{j}r_{i}^{T})\\ =r_{i}r_{j}^{T}+\frac{1}{L}r_{j}r_{i}^{T} \end{array}$$

where ${\hat{r}}_{i}$, $r_{i}$, $y_{i}(t)$ denote the ith column of ${\hat{R}}_{Y}$, the ith column of $R_{Y}$ and the ith element of y(t), respectively.

Then, the covariance matrix of Δr and $\Delta r^{*}$ is given by

(29) $$\begin{array}{cc} C & =\mathrm{cov}(\Delta r,\Delta r^{*})≜E\{(\hat{r}-r){\left({\hat{r}}^{*}-r^{*}\right)}^{H}\}=E\{\hat{r}{\left({\hat{r}}^{*}\right)}^{H}\}-rr^{T}\\ & =\left[\begin{array}{lllll} \frac{1}{L}r_{1}r_{1}^{T} & & \frac{1}{L}r_{2}r_{1}^{T} & \cdots & \frac{1}{L}r_{M}r_{1}^{T}\\ \frac{1}{L}r_{1}r_{2}^{T} & & \frac{1}{L}r_{2}r_{2}^{T} & \cdots & \frac{1}{L}r_{M}r_{2}^{T}\\ \vdots & & \vdots & & \vdots \\ \frac{1}{L}r_{1}r_{M}^{T} & & \frac{1}{L}r_{2}r_{M}^{T} & \cdots & \frac{1}{L}r_{M}r_{M}^{T} \end{array}\right] \end{array}$$

where cov(x,y) denotes the covariance matrix of x and y, and we have

(30) $$\mathrm{cov}(\mathrm{vec}(\Delta R_{r1}),\mathrm{vec}(\Delta R_{r2}))=\mathrm{cov}(G_{1}\Delta r,G_{2}\Delta r^{*})=G_{1}\mathrm{cov}(\Delta r,\Delta r^{*})G_{2}^{H}=G_{1}CG_{2}^{H}$$

From Equation (30), it can be known that $\mathrm{vec}(\Delta R_{r1})$ and $\mathrm{vec}(\Delta R_{r2})$ are dependent. Obviously, the sum of $\mathrm{vec}(\Delta R_{r1})$ and $\mathrm{vec}(\Delta R_{r2})$ still satisfies an asymptotic normal distribution

(31) $$\text{vec}(\Delta R_{r})\sim \text{AsN}(0_{M^{2}\times 1},G_{1}(\frac{1}{L}R_{Y}^{T}⊗R_{Y})G_{1}^{H}+G_{2}(\frac{1}{L}R_{Y}⊗R_{Y}^{*})G_{2}^{H}+2G_{1}CG_{2}^{H})$$

Define $W=G_{1}(\frac{1}{L}R_{Y}^{T}⊗R_{Y})G_{1}^{H}+G_{2}(\frac{1}{L}R_{Y}⊗R_{Y}^{*})G_{2}^{H}+2G_{1}CG_{2}^{H}$, and we have

(32) $$W^{-\frac{1}{2}}\text{vec}(\Delta R_{r})\sim \text{AsN}(0_{M^{2}\times 1},I_{M^{2}})$$

and

(33) $${\left\|W^{-\frac{1}{2}}\left[{\hat{r}}_{r}-(\Psi ⊙\Psi )\cdot u-\sigma^{2}\mathrm{vec}(I_{M})\right]\right\|}_{2}^{2}\sim \mathrm{As}\chi^{2}(M^{2})$$

where ${\hat{r}}_{r}=\text{vec}({\hat{R}}_{r})=\text{vec}(\frac{1}{2}Q({\hat{R}}_{Y}+J_{M}{\hat{R}}_{Y}^{*}J_{M})Q^{H})$, $\mathrm{As}\chi^{2}(M^{2})$ denotes an asymptotic chi-square with $M^{2}$ degrees of freedoms. Then, the following formula can hold with a high probability q

(34) $${\left\|W^{-\frac{1}{2}}\left[{\hat{r}}_{r}-(\Psi ⊙\Psi )\cdot u-\sigma^{2}\mathrm{vec}(I_{M})\right]\right\|}_{2}^{2}\leq \eta$$

where $\eta =\chi^{2}(M^{2})$, which can be obtained through the function $\mathrm{chi}2\mathrm{inv}(q,M^{2})$ in Matlab. Further, the formula for sparse DOA estimation is given by

(35) $$\min{\left\|u\right\|}_{1}\;s.t.\;{\left\|W^{-\frac{1}{2}}\left[{\hat{r}}_{r}-(\Psi ⊙\Psi )\cdot u-\sigma^{2}\mathrm{vec}(I_{M})\right]\right\|}_{2}\leq \sqrt{\eta }$$

It is obvious that only the matrix W is complex in Equation (35). Therefore, the operations before W are all real number operations for a low calculation complexity. The computational complexity of the operations before W is reduced by at least four times. In fact, solving Equation (35) through a second-order cone programming (SOCP) framework occupies most of the computational complexity in the proposed algorithm. Solving Equation (35) requires $O(P^{3})$ in the proposed algorithm, while it requires $O(M^{3}P^{3})$ in the L_1_-SRACV algorithm and $O(K^{3}P^{3})$ in the L_1_-SVD algorithm [5,6,20]. It is obvious that the computational complexity of the proposed algorithm is much lower than those of the other two algorithms.

The proposed L_1_-RVSKR method can be considered as the unitary L_1_-SRACV algorithm. The array covariance matrix is transformed to a real one and vectorized. By this way, a real-valued SMV model is obtained, and the calculation complexity is decreased. Moreover, the KR product is used to construct a new virtual dictionary that increases the degree of freedom.

## 4. Simulation Experiments

**Figure 1:**
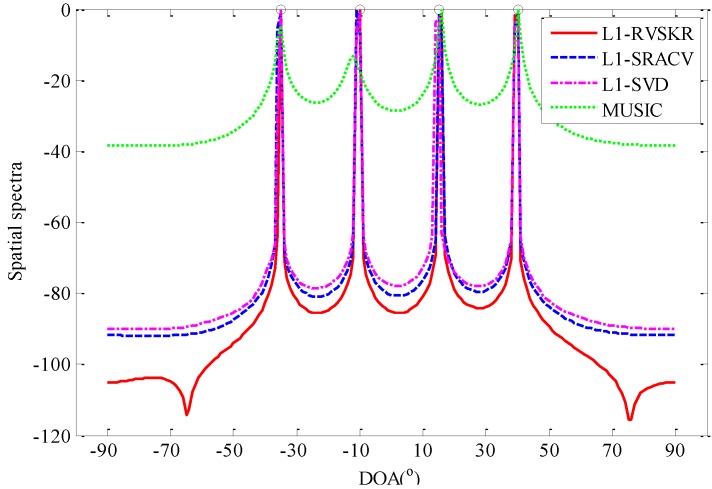
The spatial spectra for four signals with the number of sensors M=5, the number of snapshots L=500 and SNR = 0 dB.

In this section, several simulations are performed with MATLAB R2014a and CVX toolbox to verify the performance of the proposed L_1_-RVSKR algorithm. Firstly, the spatial spectra of the L_1_-RVSKR method is compared with L_1_-SRACV, L_1_-SVD, and MUSIC. Then, several experiments are performed to compare the estimation performance of different algorithms *versus* the signal-to-noise ratio (SNR), the angle interval, and the number of snapshots. Finally, the low computational complexity of the proposed algorithm is verified and analyzed by comparing the running time with the other two algorithms. We consider a ULA with λ/2 spacing in the following simulations, and the number of sensors is 8 except that shown in Figure 1 and the last simulation. The grid is divided in the range of −90° to 90° spacing 1°. The probability is set to be q=0.999 to achieve the parameter η, and the average value of the M−K smallest eigenvalues of ${\hat{R}}_{Y}$ is used as $\sigma^{2}$ in the L_1_-RVSKR and L_1_-SRACV algorithms.

### 4.1. The Spatial Spectra Comparison

**Figure 2:**
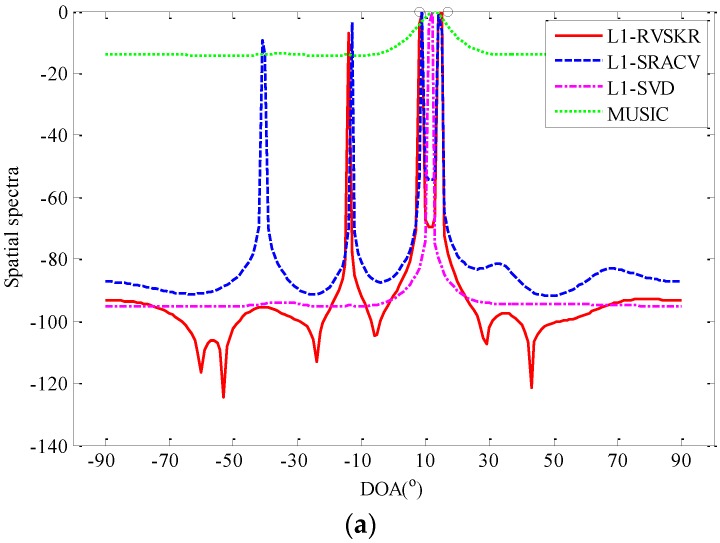

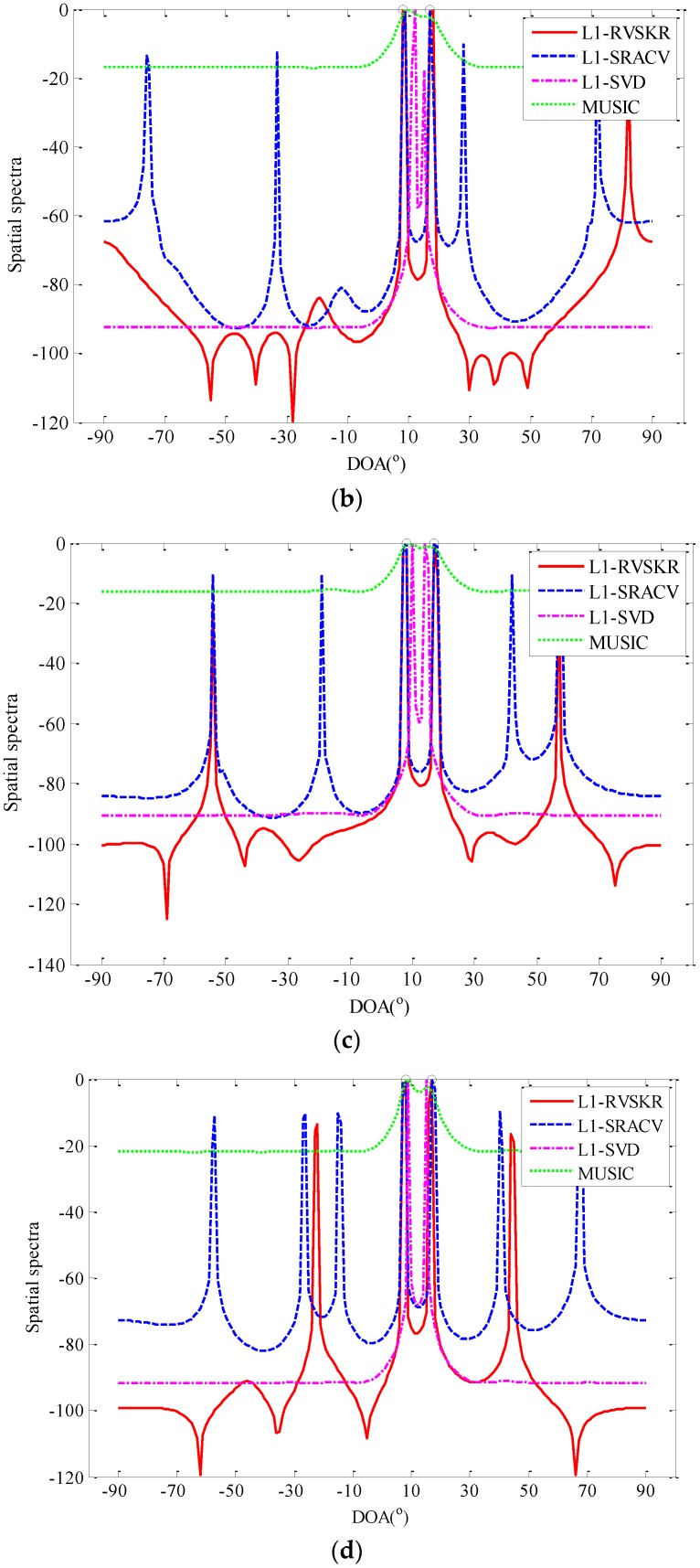

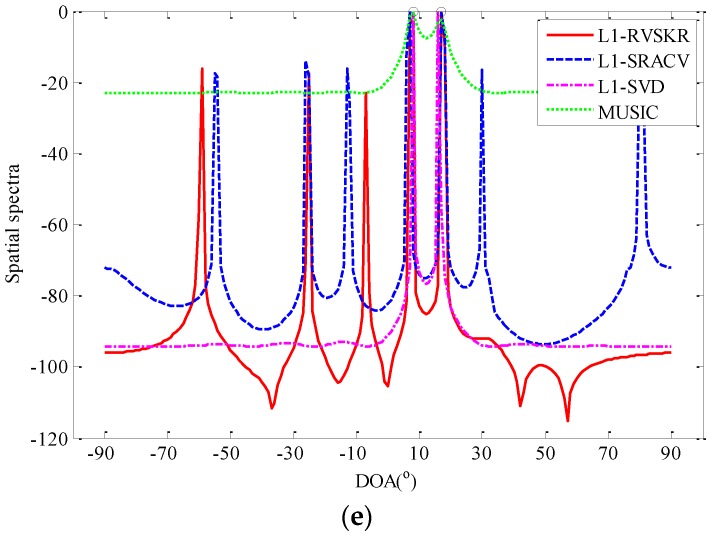
The spatial spectra for two signals with the number of sensors M=8 and the number of snapshots L=300 : (**a**) SNR = −8 dB; (**b**) SNR = −6 dB; (**c**) SNR = −4 dB; (**d**) SNR = −2 dB; (**e**) SNR = 0 dB. DOA: direction of arrival; RVSKR: real-valued sparse DOA estimation algorithm based on the KR product; SRACV: sparse representation of array covariance vectors; SVD: singular value decomposition; MUSIC: multiple signal classification; SNR: signal-to-noise ratio.

In this simulation, the spatial spectra of the L_1_-RVSKR algorithm is compared with L_1_-SRACV, L_1_-SVD, and MUSIC. Consider four uncorrelated far-field narrowband signals arriving at the array from directions [−35°,−10°,15°,40°]. The number of sensors is 5, and the number of snapshots is 500. Figure 1 shows the spatial spectra of different methods with SNR = 0 dB. It can be seen from Figure 1 that the spatial resolution of the L_1_-RVSKR algorithm is better than those of the other algorithms, and it has no pseudo-peaks in such a simulation condition. In Figure 2, we consider two uncorrelated far-field narrowband signals arriving at the array from directions 8° and 17°. The number of sensors is 8, and the number of snapshots is 300. Figure 2 shows that the L_1_-RVSKR algorithm can distinguish each signal in different SNRs. Several pseudo-peaks appear with the L_1_-RVSKR and L_1_-SRACV algorithms when SNR is high, but the pseudo-peaks are lower than that of the real estimated DOA. With the decrease of the SNR, there is only one pseudo-peak of the L_1_-RVSKR algorithm left, which is less than that of the L_1_-SRACV algorithm. Moreover, the L_1_-SVD and MUSIC algorithms cannot distinguish the signals with low SNR, although they have no pseudo-peaks.

According to the above simulation results, the L_1_-RVSKR and L_1_-SRACV algorithms are easy to produce pseudo-peaks because they are realized by L_1_-norm minimization. The problem can be solved by the idea of a weighted L_1_-norm constraint [21,22].

### 4.2. The Estimation Performance versus SNR

In this section, the estimation performance of different methods *versus* the SNR is compared by an experiment. Firstly, we define the root mean square error (RMSE) as follows:

(36) $$\mathrm{RMSE}=\sqrt{\sum_{n=1}^{N}\sum_{k=1}^{K}\frac{{\left({\hat{\theta }}_{kn}-\theta_{k}\right)}^{2}}{KN}}$$

where N denotes the number of independent Monte Carlo simulations, and ${\hat{\theta }}_{kn}$ is the estimated value of $\theta_{k}$ in the nth simulation.

**Figure 3:**
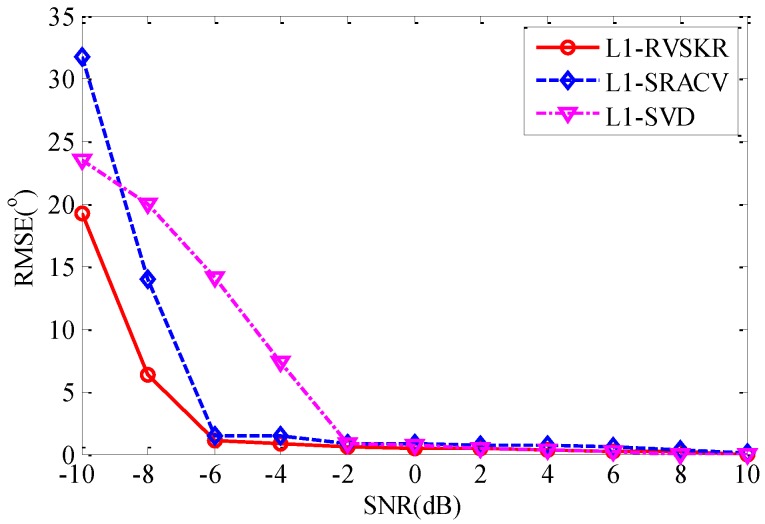
Root mean square error (RMSE) of DOA estimation *versus* SNR.

Similarly, we consider two uncorrelated far-field narrowband signals from directions 8° and 17°. The number of snapshots is 300. The SNR varies from −10 dB to 10 dB with 2 dB steps. For each SNR, 50 Monte Carlo simulations are performed to verify the performance of the proposed method. The simulation result is shown in Figure 3. It is shown that the RMSEs of the three algorithms decrease with the increase of the SNR. The RMSE of L_1_-RVSKR is less than those of the other two methods when the SNR is less than −6 dB. It is declared that the proposed L_1_-RVSKR algorithm can achieve better performance than the other two methods with low SNR.

### 4.3. The Angle Resolution Capability

**Figure 4:**
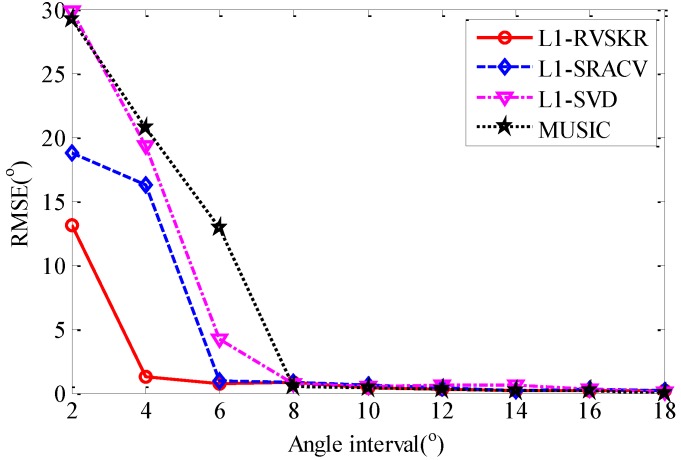
RMSE of DOA estimation *versus* the angle interval.

In this section, the angle resolution capabilities of different algorithms are compared. We suppose that there are two uncorrelated signals whose directions are 8° and Δθ+8°. The angle interval Δθ varies from 2° to 18° with a step of 2°. For each angle interval, 50 Monte Carlo simulations are performed to compare the angle resolution capability of the proposed method with MUSIC, L_1_-SVD, and L_1_-SRACV. The SNR is set to 0 dB, and the number of snapshots is 300. Figure 4 shows the comparison of the angular resolution of different methods. It can be seen from Figure 4 that the RMSEs of the four methods decrease with the increase of the angle interval. The RMSE of L_1_-RVSKR decreases significantly when the angle interval is smaller than 4°, and the performance of L_1_-RVSKR is superior to the other methods when the angle interval is smaller than 6°. This simulation illustrates that the resolving ability of the proposed method is superior to those of the other three methods.

### 4.4. The Estimation Performance versus the Number of Snapshots

**Figure 5:**
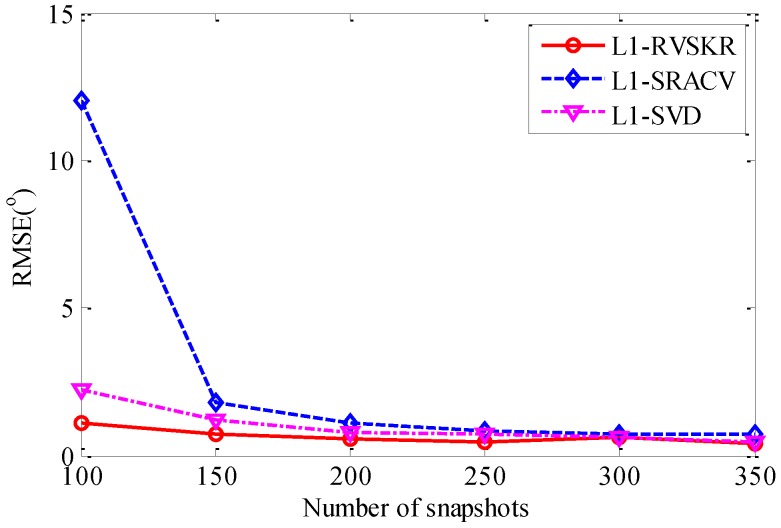
RMSE *versus* the number of snapshots.

In this section, we analyze the relationship of the RMSE of the DOA estimation and the number of snapshots in the case of SNR=0dB. Consider two uncorrelated far-field narrowband signals impinging on the array from directions 8° and 17°. The number of snapshots varies from 100 to 350 with a step of 50. For each number of snapshots, 50 Monte Carlo simulations are performed to verify the performance of the proposed method. It can be seen from Figure 5 that the RMSEs of the three methods decrease as the increase of the number of snapshots. It is illustrated in this simulation that the RMSE of the L_1_-RVSKR algorithm is smaller than the other two methods, and the L_1_-RVSKR algorithm has a better performance with a small number of snapshots.

### 4.5. The Algorithm Complexity Analysis

**Figure 6:**
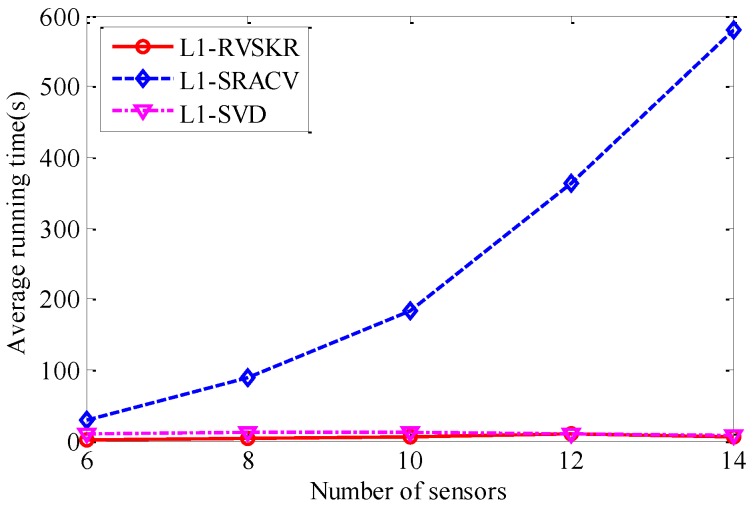
The average running time *versus* the number of sensors.

In this section, we perform 50 Monte Carlo simulations to verify the low computational complexity of the proposed L_1_-RVSKR algorithm. The average running time of the three algorithms with different numbers of sensors is shown in Table 1 and Figure 6. It is shown that the running time of L_1_-RVSKR is the shortest among the three algorithms. The proposed L_1_-RVSKR algorithm runs much faster than the other two algorithms and has a higher computational efficiency. Therefore, L_1_-RVSKR outperforms the other two algorithms with low computational complexity according to the above analysis and simulation results.

## 5. Conclusions

In this paper, we propose a real-valued sparse DOA estimation algorithm by using the KR product. The sparse model of the array covariance matrix is transformed to a real-valued one via a unitary transformation. Therefore, the calculated amount is reduced. Moreover, the vectorization is made to transform the real-valued MMV model to a SMV one. The array aperture is extended, and the estimation accuracy is improved by using the KR product. Finally, the idea of a SRACV is utilized for DOA estimation. The simulation results show that the proposed method can achieve better performance than L_1_-SVD and L_1_-SRACV with a low SNR, a small angle interval, and a small number of snapshots. The proposed method also improves the computational efficiency. However, the algorithm cannot deal with an underdetermined DOA estimation because the sparse model used is a model of SMV. We will work on this aspect in the future.

## Figures and Tables

**Table 1:** The running time *versus* the number of sensors.

Number of Sensors	L_1_-RVSKR	L_1_-SRACV	L_1_-SVD
6	3.2773 s	36.9106 s	12.9263 s
8	3.4291 s	89.6315 s	12.2859 s
10	5.8132 s	183.1054 s	12.3405 s
